# An Effective Gaze-Based Authentication Method with the Spatiotemporal Feature of Eye Movement

**DOI:** 10.3390/s22083002

**Published:** 2022-04-14

**Authors:** Jinghui Yin, Jiande Sun, Jing Li, Ke Liu

**Affiliations:** 1 School of Information Science and Engineering, Shandong Normal University, Jinan 250399, China; jinghuiyin2019@hotmail.com (J.Y.); liuke@sdnu.edu.cn (K.L.); 2 School of Journalism and Communication, Shandong Normal University, Jinan 250399, China; lijingjdsun@hotmail.com

**Keywords:** biometric recognition, behavior characteristics, gaze identification, recording duration, metric learning

## Abstract

Eye movement has become a new behavioral feature for biometric authentication. In the eye movement-based authentication methods that use temporal features and artificial design features, the required duration of eye movement recordings are too long to be applied. Therefore, this study aims at using eye movement recordings with shorter duration to realize authentication. And we give out a reasonable eye movement recording duration that should be less than 12 s, referring to the changing pattern of the deviation degree between the gaze point and the stimulus point on the screen. In this study, the temporal motion features of the gaze points and the spatial distribution features of the saccade are using to represent the personal identity. Two datasets are constructed for the experiments, including 5 s and 12 s of eye movement recordings. On the datasets constructed in this paper, the open-set authentication results show that the Equal Error Rate of our proposed methods can reach 10.62% when recording duration is 12 s and 12.48% when recording duration is 5 s. The closed-set authentication results show that the Equal Error Rate of our proposed methods can reach 5.25% when recording duration is 12 s and 7.82% when recording duration is 5 s. It demonstrates that the proposed method provides a reference for the eye movements data-based identity authentication.

## 1. Introduction

Biometrics recognition is a kind of technology that uses human biometrics for identity recognition. The commonly used biometric features are face, iris, and so on. In contrast to these static biometrics, eye movement is a behavioral biometric, recorded by eye trackers. The eye is directly controlled by the brain, and its behavior is the fastest response to changes in the environment. This makes eye movement data difficult to fake. Therefore, eye movement recognition is a highly secure identification technology.

We note that as early as 2004, Kasprowski P [1] used only 8-s recordings of eye movements during training and testing. In their paper, they mentioned that “the main problem with developing stimulation is to make it short and enriched. Eye movement recordings should not exceed 10 s”. However, later researchers seemed to have overlooked this important issue. Jia S. [2], Komogortsev O. V. [3], and Abdelwahab A. [4] used eye movement recordings of 40 s, 60 s, and 95 s, respectively. Long duration of eye movement recording can certainly bring more information and make the recognition results more accurate. However, it also reduces the recognition efficiency, and in some scenarios, users have low tolerance for recognition time. Such a long consumption is obviously unacceptable to users.

In order to achieve authentication on shorter eye movement recordings, we summarize the existing methods. Various studies have been dedicated to improving the performance of eye movement recognition. According to the extracted eye movement recognition features, they can be divided into three categories: frequency domain-based methods [1,5], statistical-based methods [6,7], and spatial-based methods [8,9]. The method based on frequency domain is the earliest method applied in the field of eye movement recognition [1]. The basic principle of frequency-domain-based methods is to perform frequency-domain analysis of eye movement recordings in the horizontal and vertical directions, respectively. Various types of frequency domain analysis methods are applied in the research, such as cepstrum, wavelet transform, and so on. A typical frequency-domain based approach was done by Kasprowski P. et al. [1], who used a Support Vector Machine (SVM) to implement authentication through the cepstrum. However, this method based on the frequency domain has some shortcomings. For example, the frequency domain analysis requires the eye movement recording to be stable, and the design of the stimulation material is required. In addition, the high-frequency noise caused by blinking and other noises generated by devices seriously pollutes the obtained frequency domain information.

Statistical-based methods classify the eye movement recordings into fixation and saccade. Then, statistical characteristics such as the average number of fixation points, average fixation time, and average saccade speed are counted [10]. Researchers distinguish between different identities by the individual information contained in these statistical characteristics. Daniel L. et al. [6] recorded eye movement data while watching and typing. They defined the number of fixation points, fixation time, average saccade speed, and some other statistical features for identification, and use these statistical features to achieve identification. Statistics-based methods are widely used in eye movement recognition, but statistical features require a large amount of data to obtain stable distributions. In addition, statistical features need to accurately classify fixation and saccade, which is greatly affected by the classification results. Therefore, statistical features have high requirements for classification algorithms.

The spatial-based method maps the eye movement recordings to a two-dimensional space by means of density maps and trajectory maps, and can obtain the spatial distribution information of eye movements. Rigas I. et al. [8] calculated the density map of all fixation points in each eye movement recording, and averaged the density maps of multiple fixation points obtained in the same eye movement recording. Finally, they obtained the recorded feature center. The disadvantage is that due to the huge difference in the spatial distribution of fixation and saccade, placing fixations and saccade in the same space may cause the spatial information between fixation and saccade to interfere with each other. Their overlapping in space also causes some parts of fixation and saccade to be obscured and cause information loss.

The duration of eye movement recording used in the existing research is too long, which reduces the recognition efficiency and user experience. In order to determine the appropriate duration of eye movement recording, this paper analyzes the concentration changes of the subjects during the recording of eye movement data. We use the deviation angle of the fixation point relative to the stimulus point on the screen to represent the subject’s concentration [11] to the stimulus material. Since blink frequency is related to concentration [12], changes in blink count over time are also taken into account. We observe that the subjects’ gaze shifted gradually over time. Deviation from the central visual field at the 5th and 12th seconds, indicates that the subject’s concentration has deviated from the stimulus point at this time. Therefore, this paper recommends that the duration of eye movement recordings should be less than 12 s. Based on existing datasets, we construct datasets with eye movement recordings of 5 s and 12 s.

Take into account that the shortening of the duration of the eye movement recording means less information. The information in eye movement recordings is still a kind of visual information in nature, which means it has rich spatial information. Spatiotemporal information features such as distance change and direction change between gaze points are extracted. The saccade trajectory in each eye movement record is mapped to a saccade distribution map, and the spatial distribution characteristics of the saccade are extracted. We combine these two features to more fully exploit the information contained in the eye movement recordings. In both open-set authentication and closed-set authentication experiments, the features proposed in this paper have obtained better authentication results on the datasets constructed in this paper.

We propose an authentication method based on eye movement spatiotemporal features, in which the eye movement authentication can be implemented based on the data with much shorter recording duration. The contributions of this paper are as follows:

1. In this paper, the deviation angle of the gaze point relative to the screen stimulus point is used to represent the user’s concentration to the stimulus during the eye movement data recording process. By experimenting on a dataset containing only fixation, we found that the subject’s concentration deviated from the stimulus at 5 and 12 s. Therefore, we recommend that the duration of the eye movement recording should be less than 12 s. On this basis, we constructed 5 and 12 s eye movement datasets based on an existing dataset;
2. The spatiotemporal information is extracted, including the distance change and direction change between the gaze points and the spatial distribution information of saccade. They are combined as a feature representation of identity. We use metric learning to authentication task on the basis of the eye movement recording duration and the constructed dataset proposed in this paper. We finally achieve better results than other methods.

## 2. Related Work

### 2.1. Eye Movement Recording Duration and Concentration

Most of the eye movement researches did not consider the problem that the long duration of eye movement recording reduces people’s interest. Whether people are interested in visual material can be judged by whether the visual material is located in the fovea. When a person looks at an object, an image of the object is projected onto the retina, which consists of photoreceptor cells that convert light into signals that are then transmitted to the brain via the optic nerve. The density of such photoreceptor cells on the retina is uneven, being denser in the center of the retina than in the periphery. This clustering results in changes in vision, with the most detailed vision obtained when the object of interest falls in the center of the retina. This area, called the yellow spot or fovea, covers a viewing angle approximately two degrees in diameter [13,14]. Outside this area, vision declines rapidly. Eye movements are performed to reorient the eye so that the object of interest falls on the fovea and the highest level of detail can be extracted. When the eyes fixate on something for a period of time, this eye state is called fixation. During this time, the brain analyzes the images projected on the fovea. Therefore, if a person’s gaze area is projected in the fovea area, it means that the person is interested and has concentrated attention in the gaze area. Conversely, the person is disinterested and distracted from the gaze area [15,16,17].

Christoforou et al. [11] and Maffei et al. [12] found that the offset distance of the subject’s gaze point relative to the stimulus material, which we call distraction distance, and the number of blinks were positively correlated with the subject’s degree of distraction. Longer distraction distances or more blinks represent less concentration on the target. We count distraction distance and the number of blinks on a fixation dataset that does not include saccades. This dataset is recorded by simply displaying a fixed point on the screen as a stimulus material to elicit the subject’s fixation behavior. Since the positions of the human eye and the stimulation material on the screen are fixed, the angle between the sight line and the line connecting the eyeball to the stimulation material, which we call the distraction angle, is positively related to the distraction distance. We can use the distraction angle to represent the degree of distraction. When the distraction angle is greater than about 1 degree, the position of the stimulation material is located outside the fovea. That means the person does not pay enough attention to the stimulation material and has lost interest in the stimulation material. Our experiments show that distraction angle gradually spreads out over time, with a peak close to 1 degree at 5 s. By quadratic fitting of the curve of distraction angle versus time, we find that the fitted distraction angle reaches 1 degree at 12 s. Therefore, this paper recommends that the duration of eye movement recording should be less than 12 s. Based on existing datasets, we construct a dataset with eye movement recording durations of 5 s and 12 s.

### 2.2. Stimulus Material

Stimulus materials are used to elicit eye movement behaviors such as saccades and fixation. The existing stimulation materials mainly include designed jump dots, random jump dot, text, video, etc. We count the stimulus materials used in existing work, and the results are shown in Table 1. The influence of different stimulus materials on eye movement data can be distinguished in two aspects: First, whether it causes the subjects to have a problem, called the learning effect [1]. Learning effect means that when the subject repeatedly watches the same stimulus material, the subject produces memory of the stimulus material, so that the subject’s interest in it gradually decreases [1]. Second, whether there is a requirement for the subjects to gaze at the designated area.

The designed jump dots display a jump point at a specified location and time. The user is required to follow the jump dots for gaze. The advantage of designed jump dots is that they can elicit fixation of a specified duration and saccades of a specified duration and amplitude [18]. The drawback is that the user has no free will to move the eye in response to the jump dot. This results in the loss of all information from the brain, resulting in biometric information loss [19]. In contrast, random jump dots do not elicit fixations and saccades with the specified duration or amplitude, but can elicit fixations and saccades with a greater variety of directions and lengths. Since random jump points cannot be memorized, the learning effect can be better relieved. Text refers to rendering a piece of text on the screen. Unlike jump dots, the user is not required to view a designated area. Therefore, users can browse the text content at will without being limited by time and text space position. This better simulates brain activity during stimulation. However, it has its own drawbacks, as it can cause a strong learning effect [1]. This problem can be solved by presenting a different text to the user each time the simulation is performed, but care should be taken that the different text presented should have the same difficulty level, so that the number of glances and fixations does not change drastically [19]. As a temporal sequence, video has a wide range of visual stimuli, which can relieve learning fatigue well. At the same time, video is the same as text in terms of behavioral constraints, and does not impose restrictions on the free will of users.

### 2.3. Eye Movement Features

In 2004, eye movement was proposed as a biological feature [1]. Subsequent studies have mainly focused on the feature representation of eye movement individual information. The main methods proposed in this domain mainly include frequency domain-based methods, statistics-based methods, and spatial-based methods. Statistics on related work published from 2004 to 2019 can be found in [30].

#### 2.3.1. Frequency Domain-Based Methods

In methods based on frequency domain analysis, eye movement recordings are processed from both horizontal and vertical directions [31]. Common frequency domain analysis methods that can be used for eye movement recording include wavelet transform [22], cepstrum [1], and Mel frequency cepstral coefficients [5,32,33], etc. Since the eyes are directly controlled by the brain, they are the fastest response to environmental changes [1]. When eye behavior is not constrained, the speed and direction of eye movement are not fixed. The eye movement recordings thus obtained are time-varying signals and do not have stationarity. In order to perform frequency domain analysis on eye movement recordings, scholars presented stimulus points [1,5,22] on the screen in a fixed sequence, and asked subjects to fixate on these stimulus points to induce a fixed pattern of eye movement behavior. By this way, the eye movement recordings are generally stabilized. At the same time, because the human eye inevitably blinks and has other behaviors, the eye movement recording is prone to extraneous noise. These extraneous noises usually appear in the spectrum in the form of high-frequency noise, which are easily confused with saccades and cause serious information pollution in the frequency domain [24].

#### 2.3.2. Statistical-Based Methods

Because the frequency domain analysis has problems such as signal stability requirements and sensitivity to extraneous noise. Statistics-based methods [6,24,34] have gradually become the main eye movement recognition methods. Since it is difficult to compare between raw eye movement recordings [1], statistics-based methods use classification methods to classify eye movement recordings into eye movement behaviors such as fixation and saccade [10,18]. The basic principle of the eye movement behavior classification algorithm is that the speed of fixation and saccade has a immense difference. In addition, there were differences in the duration of fixation and saccades. Therefore, fixation and saccade can be screened by setting the speed threshold and the duration of the eye movement recording segment. The classification process can filter extraneous noise caused by blinks and errors inherent in the eye tracker system.

After the fixation and saccade clips are obtained, some artificially designed statistical indicators are extracted from them. These metrics are used to express statistical differences in eye movement behaviors among different populations. For example: average duration of fixation, average duration of saccades, average number of fixations in a saccade, etc. [6,24,34]. These indicators can play different roles in eye movement recognition, so they are usually given different weights for feature selection [7,35]. In the study of Rigas et al. [36], thousands of statistical indicators were proposed. Statistical features can be used for any eye movement data and have broad application prospects. However, these statistical features require a large amount of data to obtain stable distributions. In addition, artificially designed features can cause some information to be lost.

#### 2.3.3. Spatial-Based Methods

Some researchers have attempted to extract spatial information from eye movement data. Rigas I. et al. [8] asked 200 subjects aged 18–44 to watch 2 videos twice, each with 4 eye movement recordings. The density maps of all fixation points in the eye movement recordings in each two-dimensional space were calculated, and the multiple density maps obtained from the same segment of eye movement recordings were averaged to finally obtain the feature centers of the recordings. Li Chunyong et al. [9] obtained eye movement recordings in a visual search task, using images to restore the path of recording eye movements in space. Texture features are extracted from eye-tracking trajectories using multi-channel Gabor Wavelet Transform (GWT). A SVM classifier was used for biometric identification and verification. Then, this feature is improved in [37] by downsampling the filtered image to preserve the spatial structure. However, these features compressed the eye-tracking data in the temporal dimension, reducing the temporal information contained in the data. They also ignored the problem of spatial information loss caused by overlapping saccades and gazes in space.

Recently, some researchers have introduced neural network [2] and metric learning [4,38] into the field of eye movement recognition, and obtained good results. The features proposed in this paper combine temporal information with more complete spatial information. By combining it with metric learning, individual information contained in eye movement data can be better utilized for recognition.

## 3. Eye Movement Recording Duration

Eye movement recognition is a way of identifying behavioral characteristics as biometrics. Behavioral data is a kind of data that has a time span. The duration of a single eye movement recording affects its amount of information. Therefore, it has a direct impact on the recognition result. However, the existing methods do not unify the duration of eye movement recordings. We count the duration of eye movement recordings used by some previous eye movement recognition methods and the results are shown in Table 2.

We find that most of the previous works used eye movement recordings with a duration of more than 60 s. This obviously reduces the efficiency of recognition and challenges the patience of users. In order to analyze the changes of people’s concentration and interest over time in the process of recording eye movement data, we perform experiments on a dataset recorded with a fixed point as the stimulus to determine a proper eye movement recording duration.

**Figure 1:**
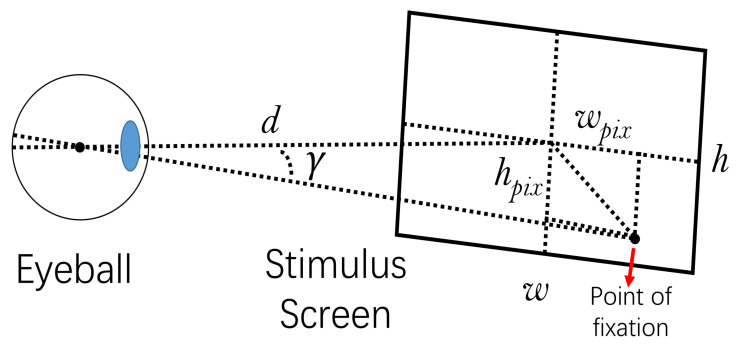
Optical geometry for gaze recording.

We denote the distraction distance (the distance between the gaze point and the stimulus point) [11] as *D* and denote the number of blinks as *P* to measure the change of the subject’s attention during the process of gaze data collection. The number of blinks is stored as NaN in the data. It has been shown that an increase in the number of blinks represents a decrease in attention [12]. We split every recording to *m* time periods. For every eye movement recordings in the time period *m*, we use Equation (1) to calculate the distraction distance *D*, and use Equation (2) to calculate the number of blinks *P*. We calculate the distraction angle γ by the average distance of each distraction distance in the time period *m* as described in Equation (3). Then we observe the trend of γ and *P* over time, and compare γ with the human central visual angle [13,14] to determine a reasonable eye movement recording duration. The eye movement recording process is shown in Figure 1. The experiments and results will be introduced in Section 5.

(1) $$D=\frac{\sum_{j=0}^{n}\left|d\left(x_{j},y_{j}\right)-T\right|}{n},$$

(2) $$P=\frac{\sum_{j=0}^{n}p}{n},$$

(3) $$\gamma =\frac{\arctan(D\cdot h/\left(h_{pix}\cdot l\right))\times 180}{\pi },$$

where *n* refers to the number of gaze points in the time period *m*, *d* refers to a gaze point in *m* and *T* is the stimulus point, *l* refers to the distance between the subject’s head and the screen, *h* is the actual height of the screen, and $h_{pix}$ refers to the pixel resolution height of the screen.

**Figure 2:**
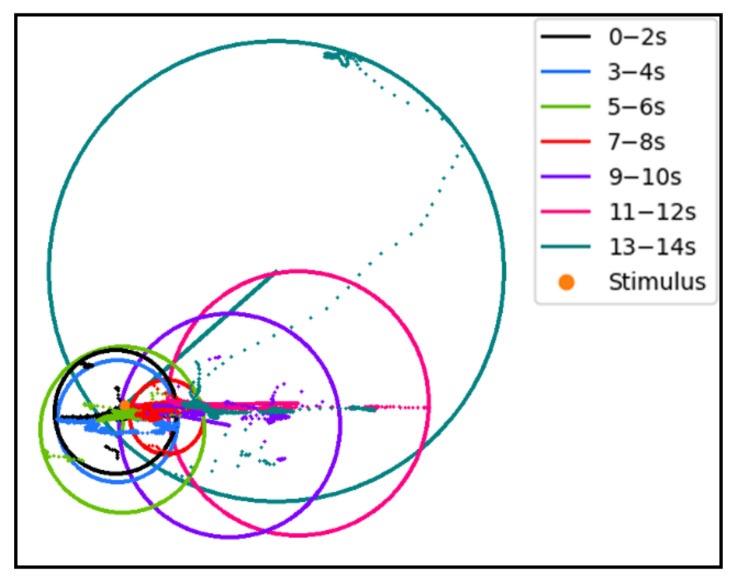
Example of an eye movement recording split into seven segments by time.

We show an example of eye movement recording in Figure 2. Gaze points in different time periods are marked with different colors. We draw the circumscribed circle of all gaze points in each time period to represent the dispersion of fixation points, and draw the line between the center of the circle and the stimulus point to represent the distance between the gaze point and the stimulus point. It shows that as time progresses, the gaze points gradually deviate from the stimulus point and become more dispersed.

## 4. Our Methods

### 4.1. Data Pre-Processing

Eye movement recordings can be split into gazes and saccades through an eye movement classification algorithm. These classification algorithms can be divided into velocity-based, dispersion-based, and region-based methods [41]. There are two kinds of velocities during eye movements, which are low velocity (<100 degrees/s) for fixation and high velocity (>300 degrees/s) for saccades. It makes the eye movement classification based on velocity simple and robust. That is why we use the velocity thresholding algorithm [3] to classify the eye movement.

The eye movement data used in this paper includes gaze angles in the *x* and *y* directions, and these data are recorded at a sampling rate of 1000 Hz. Since the method proposed in this paper mainly uses the spatial information of the eye movement data, these data needs to be converted into the form of screen coordinates in the preprocessing stage. We use the method as described in Equations (4) and (5) to get the screen coordinates:

(4) $$x_{screen}=\left(\frac{l*w_{pix}}{w}\right)tan\left(\theta_{x}\right)+\frac{w_{pix}}{2},$$

(5) $$y_{screen}=\left(\frac{l*h_{pix}}{h}\right)tan\left(\theta_{y}\right)+\frac{h_{pix}}{2},$$

where, *l* refers to the distance between the subject’s head and the screen, *w* and *h* are the actual width and height of the screen, respectively, and $w_{pix}$ and $h_{pix}$ refer to the pixel resolution width and height of the screen, respectively. $\theta_{x}$ and $\theta_{y}$ refers to the line of sight angle stored in the original data.

### 4.2. Motion Information

The gaze consists of eye movements, which is a kind of behavior. The motion information (MI) of gaze covers the temporal and spatial features extracted from the trajectory of eye movement. Hence, the MI is used as the feature to represent the gaze.

We set a vector *b*, whose starting and ending points are the coordinates of two gaze points that are adjacent in time. We use *b* to model the spatial changes of adjacent fixation points. As we all know, a vector can be determined by only two factors: the vector length and the vector direction. Therefore, the spatial change of the gaze points in the eye movement recording can be represented by the length and the direction of *b*. We respectively calculate the distance between adjacent gaze points and the change of direction to extract the spacial motion information. Suppose that two adjacent fixation points are represented by $P_{i-1}=\left(x_{i-1},y_{i-1}\right)$ and $P_{i}=\left(x_{i},y_{i}\right)$, where $P_{i-1}$ represents the forward fixation point, and $P_{i}$ represents the latter fixation point. The calculation method of the change distance between $P_{i-1}$ and $P_{i}$ is described as Equation (6).

(6) $$L=\sqrt{{\left(x_{i}-x_{i-1}\right)}^{2}+{\left(y_{i}-y_{i-1}\right)}^{2}},$$

Let the vector a=(0,1) be the reference vector. Calculate the vector $b=(x_{i}-x_{i-1},y_{i}-y_{i-1})$ with $P_{i-1}$ as the starting point and $P_{i}$ as the ending point. We denote the angle that the vector *a* rotates clockwise until it coincides with the vector *b* as θ to indicate the direction change of the gaze points. Suppose that one eye movement recording has *n* gaze points. θ is calculated from each pair of adjacent gaze points in an eye movement recording to get a 1×(n−1) sequence, as described in Equation (7).

(7) $$\theta =\left\{\begin{array}{ccc} \arccos(\frac{\alpha \cdot \beta }{\left|\alpha ||\beta \right|}), & \; & if\;x_{1}-x_{0}\geq 0,\\ 2\pi -\arccos(\frac{\alpha \cdot \beta }{\left|\alpha ||\beta \right|}, & \; & if\;x_{1}-x_{0}<0, \end{array}\right.$$

**Figure 3:**
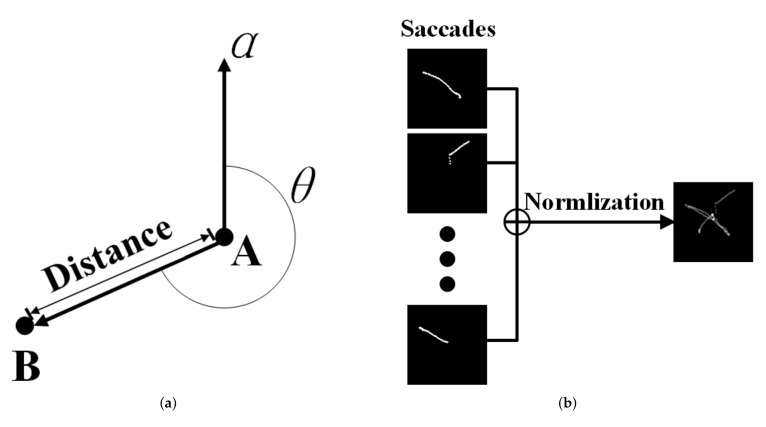
(**a**) Motion information from gaze points A to B. (**b**) Saccade distribution map.

The geometric representation of the gaze point motion information is shown in Figure 3. We fuse the distance motion feature and the direction motion feature to get a 2×(n−1) feature and normalize it with the z-score. This feature serves as a representation of the change of the spatial information of gaze points.

### 4.3. Saccade Distribution Map

In a data recording process, the subjects watch the materials on the screen by alternately making fixations and saccades, because the spatial information contained in fixation trajectory is not obvious, and the trajectory generated by each body during saccade has obvious individual information in space. In addition, the fixation trajectory may overlap the saccade trajectory in space, causing information loss. Therefore, we choose to map each saccade to a feature image, which we call the saccade distribution map (SDM).

Suppose the saccade trajectory is a two-dimensional spatial coordinate sequence $T_{i}=\left\{\left(x_{1},y_{1}\right),\left(x_{2},y_{2}\right),\cdots ,\left(x_{n},y_{n}\right)\right\}$. We calculate the maximum horizontal and vertical distance, which corresponds to the maximum width and maximum height of the saccade trajectory in space. The sequences whose maximum width or maximum height are too short are then discarded. Then, each saccade coordinate point is mapped to a 1680×1050 image and all the images are scaled to a size of 128×128. These images represent the spatial distribution of the corresponding saccade trajectory. Before entering these feature maps to our network, they are divided by their own pixel sum separately to get the probability images. The process to get a SDM is shown in Figure 3.

### 4.4. Architecture

**Figure 4:**
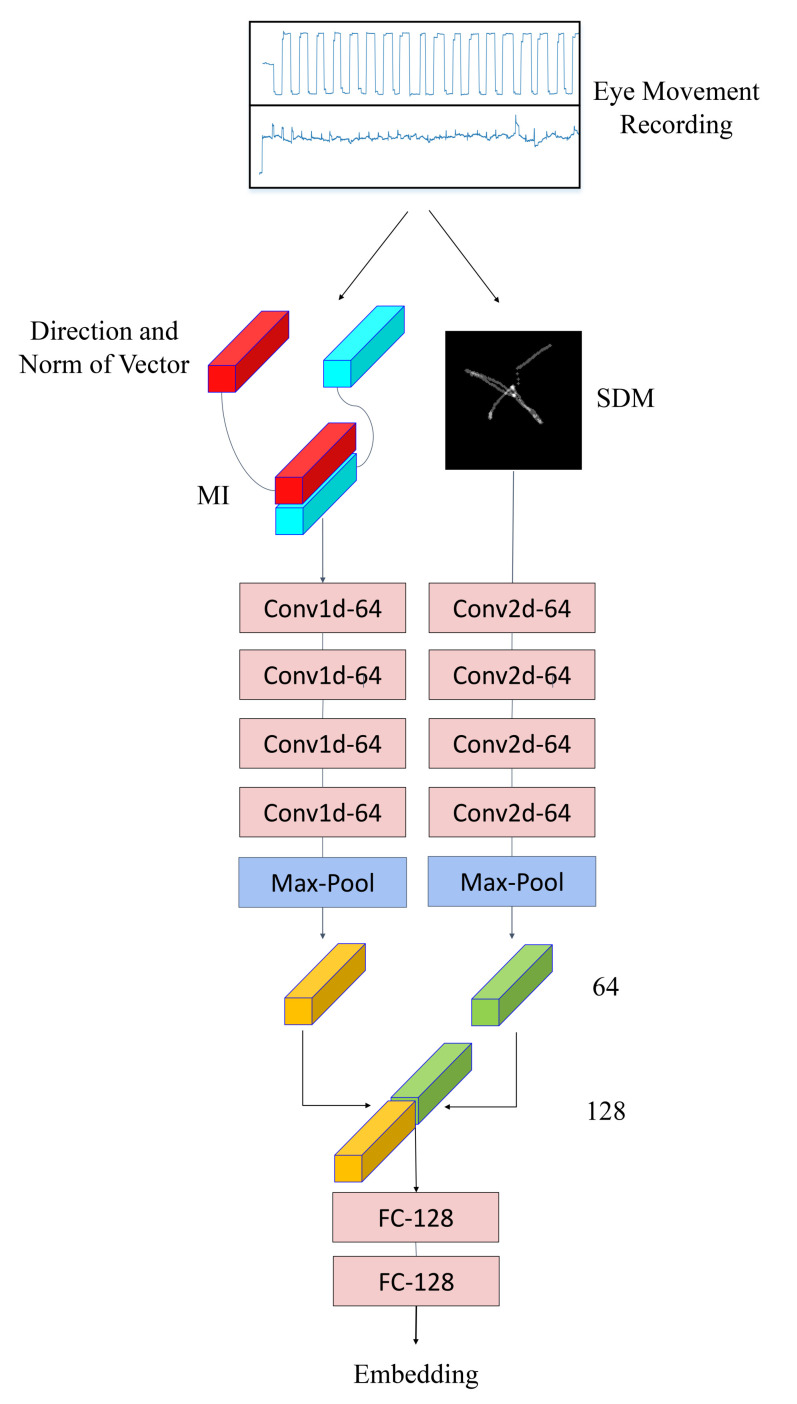
Our architecture for eye movement features extraction.

We design a network to realize feature extraction and classification. Two branch networks are designed to perform further feature extraction on MI and SDM, respectively. All pipelines are shown in Figure 4. Since saccades and fixations are not related to the position in eye movement recordings, convolutional neural network (CNN) is a good network for feature extraction. The branch network that processes SDM contains four 2D-CNNs, and each 2D-CNN has 64 filters, a kernel size of (5,5), and a dilation of 1. The purpose of using dilated convolutions is to expand the perceptive view of each filter. The branch network that processes MI contains 4 1D-CNNs, and each 1D-CNN has 64 filters, a kernel size of 7, and a dilation of 7. Each convolutional layer used the batch normalization followed by the ReLU activation function. Then, the outputs of the two branch networks are spliced and input into two fully connected layers. The fully connected layers are used for feature fusion and classification. The result obtained by the fully connected layer is flattened into a one-dimensional array, and L2 normalization is performed on it, and finally a 128-dimensional embedding feature is obtained.

## 5. Experiments

### 5.1. Platform

The operating system is Ubuntu 16.04. The hardware platform is Intel Core CPU i7-7700K 4.2GHz and 16 GB DDR4-2133 RAM. The Graphic Processing Unit is GeForce RTX 2080Ti 11 GB.

### 5.2. Datasets

The dataset used in this paper is a part of GazeBase v2.0 [23] recorded by Henry Griffith and Oleg Komogortsev et al. GazeBase v2.0, and contains 12,334 monocular eye movement recordings collected from 322 college-age participants. It has a total of nine rounds of data at different times. During each round of recording, participants finish a set of seven tasks in two consecutive sessions, including (1) gaze task (FXS), (2) horizontal saccade task (HSS), (3) random squinting task (RAN), (4) reading task (TEX), (5/6) free watching movie and video task (VD), and (7) gaze-driven game task (BLG). In the same round, each task can collect two eye movement recordings from each participant. The recording process lasted 37 months, and participants in each round were recruited from the previous round. The eye movement data is recorded using the EyeLink 1000 eye tracker at a sampling rate of 1000 Hz, including data such as the coordinates of the eye movement and pupil size. The distribution of the number of participants in GazeBase v2.0 in each round is shown in Table 3.

### 5.3. A Proper Eye Movement Recording Duration

In GazeBaseV2.0, a sub-dataset FXS recorded fixation recordings from 322 subjects by presenting stimulus points at a fixed position on the screen. The duration of these recordings is about 15 s. Since the subjects need a certain amount of time to notice the stimulus at the beginning of each recording, we removed the first second data of each recording and only used the last 14 s for our experiment.

**Figure 5:**
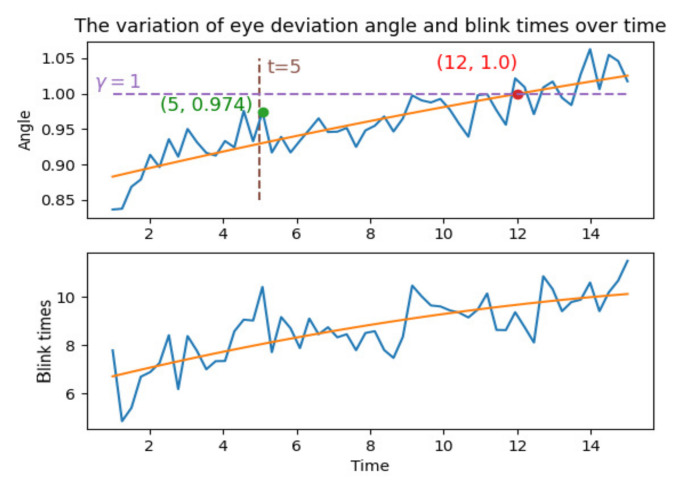
The average distance from the fixation point to the target point over time and the average number of blinks per recording.

We split all recordings into non-overlapping segments of 0.5 s, so that each recording could get 28 segments. The method described in Section 3 is performed to calculate the mean value of γ and *P* and curve fitting is performed on it. The results are shown in Figure 5. The two indicators, the distraction angle γ and the number of blinks *P* of the user in the data recording process, increased over time. According to the research in [11], this shows that the subject’s concentration when looking at a fixed position decreases with time and becomes less and less concentrated.

At the 5th second, the distraction angle γ has a peak value close to 1°. When the time reaches the 12th second, the fitted curve indicates that γ is about 1°. The radius of the viewing angle range corresponding to the fovea is about 1° [13,14]. This means that over time, the position of the target point will gradually deviate from the visual range of the fovea, which means that the subject’s concentration to the stimulus point will gradually deviate from the visual range of the central fovea. Therefore, we recommend that the duration of the eye movement recordings is no longer than 12 s. This paper chooses 5 s and 12 s as the eye movement recording duration for our experiment.

### 5.4. Authentication Results

The closed-set authentication means that each one in the test set have to be identified as one in the training dataset no matter what the match rate is, while in the open-set authentication, the one with the lower match rate than the predefined threshold will not be identified as any one in the training dataset. These are the two kinds of strategies in the field of authentication. Hence, in this section, we demonstrate our proposed method through the open-set and closed-set authentication experiments.

#### 5.4.1. Open-Set Authentication

According to whether the target identity is identified in the training dataset, the identity recognition task can be divided into two categories: closed-set recognition and open-set recognition. Closed-set recognition means that all people in testing must appear in training. Open-set recognition means that people in testing will not appear in training.

This paper focuses on open-set authentication task. In GazeBase v2.0, the number of subjects in each round of the first 9 rounds of testing is different, which means that the amount of data of the subjects is different. In order to eliminate the impact of the inconsistency of the amount of data between the experimental subjects, and to make each subject have enough data, we select 31 participants who joined in the first 8 rounds for test. For the training set and validation set, we use the method in [39] to perform 10-fold cross-validation on the other 291 subjects. In order to make the number of objects in per fold the same, we pick the first 290 subjects out from the 291 subjects. In addition, the 9th round of data is discarded in the training data. This way, for GazeBase v2.0, a total of 496 eye movement recordings are used for testing, and 1228 eye movement gaze recordings are used for the training and validation sets.

We use three sub-dataset, HSS, RAN, and TEX, in GazeBase v2.0 to reconstruct the new datasets. In accordance with the eye movement recording duration recommended in Section 5.3, we split each recording into sub-sequences of 5 s to form new datasets, HSS5, RAN5, and TEX5, and 12 s to form new datasets HSS12, RAN12, and TEX12. Due to the recording duration in TEX being 60 s, which is shorter than 100 s in HSS and RAN, we only take the first 60 s of data to use in our experiment. Therefore, for HSS12, RAN12, and TEX12, each reconstructed sub-dataset contains 6190 training recordings and 2480 test recordings. For HSS5, RAN5, and TEX5, each reconstructed sub-dataset contains 14,856 training recordings and 5952 test recordings.

In our training stage, the learning rate of our model is set to $1\times 10^{-4}$, the max epoch is 100,000, the optimizer is AdamW, and the weight decay is set to $1\times 10^{-4}$. We use MultiSimilarity Loss to measure the difference between the positive category and the negative category. The parameters of MultiSimilarity Loss are set to default. At each training step, we randomly sampled 8 subjects and 4 trajectory from each subject.

**Figure 6:**
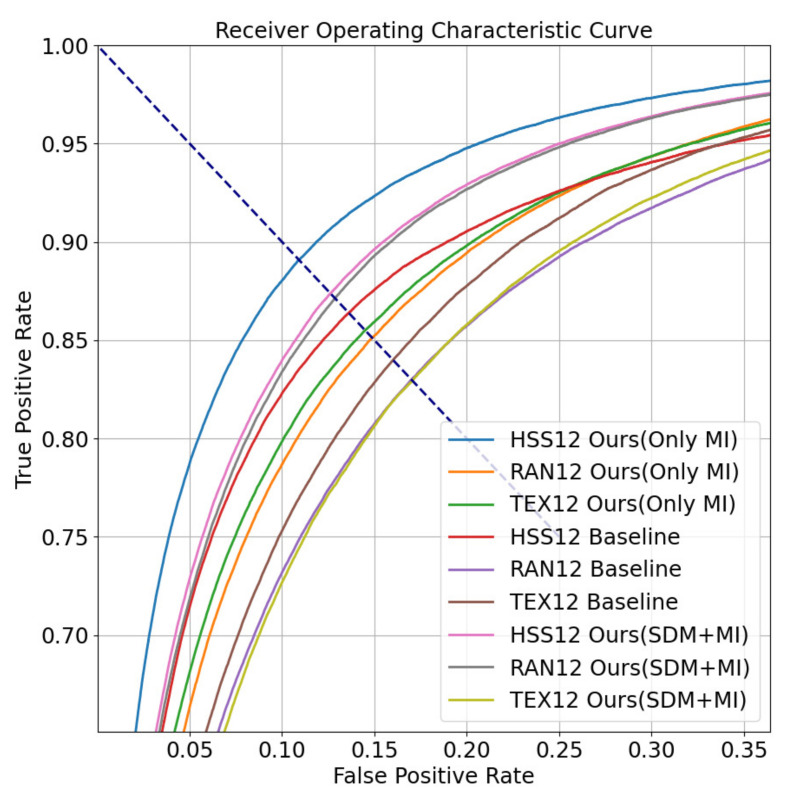
The ROC curve of our open-set authentication results when recordings last 12 s.

In the testing stage, we use cosine similarity to measure the similarity between two embeddings, and set a threshold to indicate whether the two samples belong to the same subject. We use Equal Error Rate (EER) and Receiver Operating Characteristic (ROC) curve to measure the effectiveness of the authentication model. Take dataset HSS12 as an example, we paired samples of the same subject to form genuine pairs and we got 97,960 genuine pairs. We pair the first half of the samples of each subject with the back half samples of other subjects to form the imposter pairs, and a total of 1,488,000 imposter pairs are obtained. Genuine pairs account for 6.17% of all pairs. The ratio of genuine pairs to imposter pairs is about 15:1. The results of our method are shown in Table 4 and Table 5. The best results are marked in bold. The ROC curve is shown in Figure 6. The dott line represents the EER.

In Table 4, the baseline method of [39] originally used an about 100 s eye movement recording as a single identity representation. Its EER was greatly reduced to 13.61% on HSS12, 17.06% on RAN12 and 16.07% on TEX12, which contain 12 s recordings reconstructed in this paper. Since the baseline method only used velocity data to extract features, it lost part of the information. Compared with the features of the baseline methods, the features proposed in this paper contain richer effective information, so we can get better authentication results. The EER of the method proposed in this paper reaches 10.62% on HSS12, 12.90% on RAN12, and 14.49% on TEX12. The reason why the result of Ours (SDM+MI) is worse on HSS than Ours (Only MI), but better on RAN is that the saccades on HSS overlap each other spatially. However, there is no overlap on RAN and RAN has more saccades direction. In TEX12, the effect of Ours (MI) is increased to 14.49% compared to the baseline, but the effect of Ours (SDM+MI) is even worse. This is because the position of the text stimulus materials on the screen is constantly changing when recording the eye movement data in TEX, resulting in a large difference in the location of the gaze point between different time periods. SDM refers to the spatial distribution of saccade trajectories in eye movement recordings, represented by a probability density map. SDM is suitable for eye movement data with stable location distribution of stimulus material and scattered saccade distribution with little overlap.

**Figure 7:**
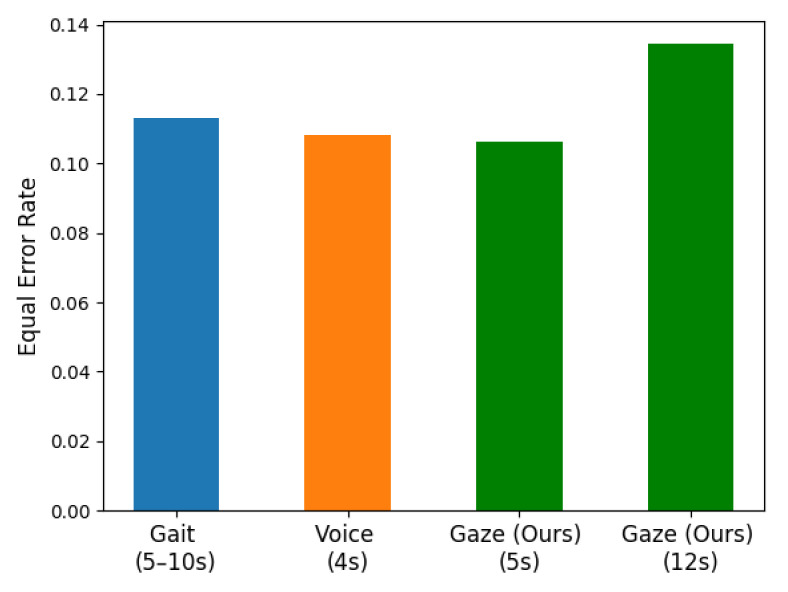
The EER of some dynamic biometrics with shorter recording.

We also experimented with the baseline method and the effect of our proposed method when the eye movement recording duration is only 5 s. The results are shown in Figure 7. The EER of baseline method is 14.98% on the 5-s dataset HSS5, 20.17% on RAN5, and 18.89% on TEX5, which we reconstructed. The EER of the method proposed in this paper reaches 12.48% on HSS5, 17.29% on RAN5, and 16.41% on TEX5. The average EER decrease of our method compared to the baseline is about 17.71%. The experimental results of all methods have different degrees of decline and the recognition effect on the HSS has the smallest decline. The experimental results of our method show that on HSS5, the EER can still reach 12.48% at the best, which is still less than the baseline result 13.61% on HSS12.

We also compare the identification effects of eye movement features and other dynamic biological features when the data time duration is short, as shown in Figure 7. In 2020, the EER of the gait recognition method proposed by Alobaidi et al. using 5–10 s of gait data for authentication is preferably 11.32%. The speaker recognition method proposed by Al-Karawi et al. has an EER of 10.8% when using voice data with a duration of only 4 s for authentication. The method proposed in this paper achieves 12.48% and 10.62% EER when using eye movement recordings with a duration of 5 s and 12 s, respectively. The identification effect is similar to that of other dynamic biometrics, indicating that eye movement recognition has certain usability.

#### 5.4.2. Closed-Set Authentication

The effect of our method on closed-set authentication is also investigated. We use the test datasets from the open-set authentication as our closed-set datasets. These test datasets, which have 31 subjects data of three sub-dataset HSS, RAN, and TEX in GazeBase v2.0, are used to reconstruct our closed-set authentication datasets. In accordance with the eye movement recording duration recommended in Section 5.3, we use the test dataset to form new datasets HSS5-C, RAN5-C, and TEX5-C and 12 s to form new datasets HSS12-C, RAN12-C, and TEX12-C. Each subject has 16 eye movement data in each task, so a total of 496 eye movement tracks are used for training, validation, and testing. Since the 8-round collection process of the GazeBase v2.0 dataset is from the 1st round of collection on 13 September 2013 to the 8th round of collection on 16 May 2015, it has a large time span. In order to test the time invariance of eye movement features and make the time results better in line with the setting that the training data time is earlier than the validation data time in real applications, we initially selected the first 4 rounds of data from 31 test subjects as the training data. and the 5th and the 6th round as validation data, and the last 2 rounds as test data. In the experiments in Section 5.3, we suggest that eye movement data with a duration of less than 12 s is used as the representation of a single identity, which can improve the efficiency of recognition and improve the user experience. Therefore, for HSS12-C, RAN12-C, and TEX12-C, each reconstructed sub-dataset contains 1860 training recordings and 620 test recordings. For HSS5-C, RAN5-C, and TEX5-C, each reconstructed sub-dataset contains 4464 training recordings and 1488 test recordings. We will experiment with our closed-set authentication method on these six new datasets.

**Figure 8:**
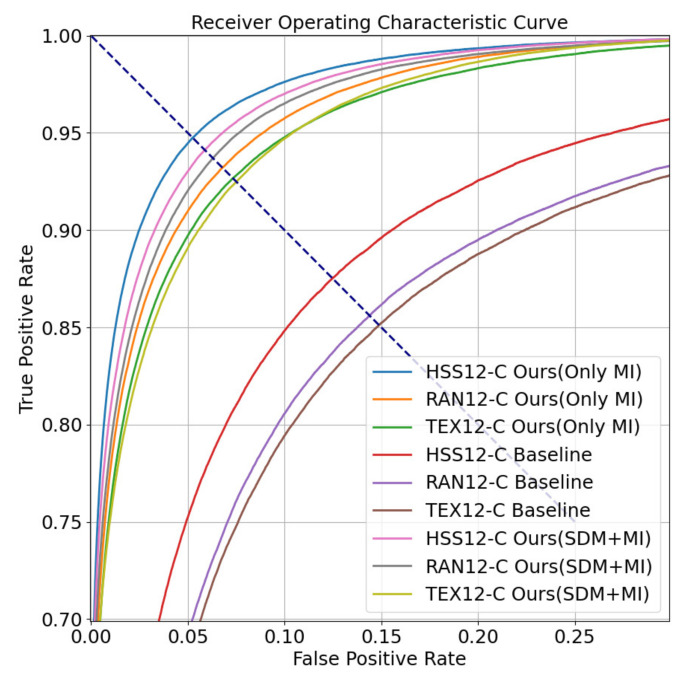
The ROC curve of our closed-set authentication results when the recordings last 12 s.

In the training stage, our closed-set authentication method is implemented by training a classifier. The output of the classifier is the ID of users. The learning rate of our model is set to $1\times 10^{-3}$, the max epoch is 20,000, the optimizer is Adam, and the weight decay is set to $1\times 10^{-4}$. We use Arcface Loss [42] as the loss function. The parameters of Arcface Loss is default. In the testing stage, we use cosine distance to measure the similarity between the embeddings output by the last fully connected layer. We also use EER and ROC curve to measure the effectiveness of the authentication model. Take dataset HSS12-C as an example, where we paired samples of the same subject to form genuine pairs and we got 5890 genuine pairs. We pair the first half of the samples of each subject with the back half samples of other subjects to form the imposter pairs, and a total of 96,100 imposter pairs are obtained. Genuine pairs account for 5.75% of all pairs. The ratio of genuine pairs to imposter pairs is about 16:1. The results of our method are shown in Table 6 and Table 7. The best results are marked in bold. The ROC curve is shown in Figure 8. The dott line represents the EER.

In Table 6, the baseline method of [24] originally used about 60 s of eye movement recording as a single identity representation. In closed-set authentication task, its EER is 12.44% on HSS12-C, 14.41% on RAN12-C, and 14.93% on TEX12-C, which contain 12 s recordings reconstructed in this paper. The EER of the method proposed in this paper reaches 5.25% on HSS12-C, 6.30% on RAN12-C, and 7.33% on TEX12-C. We also experimented the closed-set authentication with the baseline method and the effect of our proposed method when the eye movement recording duration is only 5 s. The experimental results are shown in Table 7. The EER of baseline method is 16.23% on the 5-second dataset HSS5-C, 17.76% on RAN5-C, and 20.87% on TEX5-C, which we reconstructed. The EER of the method proposed in this paper reaches 7.82% on HSS5-C, 8.21% on RAN5-C, and 9.93% on TEX5-C. The average EER decrease of our method compared to baseline is about 53.83%. The experimental results of our method show that on HSS5-C, the EER can still reach 7.82% at the best, which is still less than the baseline result 12.44% on HSS12-C. Notice that the result of Ours (SDM+MI) is still worse on HSS than Ours (Only MI), but better on RAN. This indicates that the data from HSS cause noises to SDM. In TEX12-C, the effect of Ours (MI) is increased to 7.33% compared to the baseline, but the effect of Ours (SDM+MI) is still worse. Due to the position of texts shown on the screen changing constantly, this indicates that the gaze position can influence the information included in the saccade distribution. Therefore, we can conclude that MI is useful for these three materials and SDM is only useful for RAN.

## 6. Conclusions

This paper proposes a feature to effectively express spatial information in eye movement data. The distance and direction changes between fixation points and the spatial distribution of saccades were combined as valid information in eye movement recordings. Meanwhile, this paper proposes a network for metric learning of this feature. Identity authentication is finally achieved.

In this paper, we conduct some exploratory studies on the length of recorded data used for identification. This paper presents a method to evaluate the duration of eye movement recordings for eye movement recognition. Changes in human attention were measured by whether the stimulus material was in the foveal field of vision. After the experiment, it was found that at the 5th and 12th seconds, the subject’s attention to the stimulus point deviates from the visual range of the fovea. Therefore, this paper recommends that the duration of eye movement recordings should not exceed 12 s. Appropriate eye movement recording duration can improve the efficiency of eye movement recognition and improve user experience. Based on the existing datasets, this paper reconstructs datasets with eye movement recording durations of 5 s and 12 s. The authentication method proposed in this paper achieves better results on multiple datasets constructed in this paper.

Our experimental results show that the MI proposed in this paper can achieve better results on all datasets. Our proposed SDM achieves better results only on the RAN dataset. This indicates that the distribution information of saccades is only applicable to the authentication data generated by the specific stimulus material. Considering that the research on the spatial distribution information of eye movement has not been deeply explored, it will be a valuable research direction for which kind of data is effective for eye movement distribution information.

## Figures and Tables

**Table 1:** The stimulus materials used in a previous study.

Stimulus Material	Designed Jump Dot	Random Jump Dot	Text	Video	Others
Study	[1,7,18,19,20,21,22,23]	[23,24,25]	[23,24,25,26,27,28,29]	[3,8,23,26]	[6,9,23]

**Table 2:** Duration of eye movement recordings for training and testing in a previous study.

Study	[1]	[39]	[7]	[2]	[40]	[3,9,24,25,26,29,34,39]
Duration (s)	8	15	21	30	40	60 and more

**Table 3:** The number of subjects of each round in Gazebase v2.0.

Round	1	2	3	4	5	6	7	8	9
Number of Subjects	322	136	105	101	78	59	35	31	14

**Table 4:** The results of open-set authentication when the recordings last 12 s.

Methods	HSS12	RAN12	TEX12
Baseline [39]	13.61%	17.06%	16.07%
Ours (Only MI)	**10.62%**	14.73%	**14.49%**
Ours (SDM+MI)	12.62%	**12.90%**	17.18%

**Table 5:** The results of open-set authentication when the recordings last 5 s.

Methods	HSS5	RAN5	TEX5
Baseline [39]	14.98%	20.17%	18.89%
Ours (Only MI)	**12.48%**	18.22%	**16.41%**
Ours (SDM+MI)	13.98%	**17.29%**	17.45%

**Table 6:** The results of closed-set authentication when the recordings last 12 s.

Methods	HSS12-C	RAN12-C	TEX12-C
Baseline [24]	12.44%	14.41%	14.93%
Ours (Only MI)	**5.25%**	6.79%	**7.33%**
Ours (SDM+MI)	5.88%	**6.30%**	7.51%

**Table 7:** The results of closed-set authentication when the recordings last 5 s.

Methods	HSS5-C	RAN5-C	TEX5-C
Baseline [24]	16.23%	17.76%	20.87%
Ours (Only MI)	**7.82%**	8.97%	**9.93%**
Ours (SDM+MI)	8.06%	**8.21%**	10.11%

## Data Availability

Data sharing not applicable.
